# Direct and Sensitive Electrochemical Evaluation of Pramipexole Using Graphitic Carbon Nitride (gCN) Sensor

**DOI:** 10.3390/bios12080552

**Published:** 2022-07-22

**Authors:** Yogesh M. Shanbhag, Mahesh M. Shanbhag, Shweta J. Malode, S. Dhanalakshmi, Kunal Mondal, Nagaraj P. Shetti

**Affiliations:** 1 Department of Chemistry, School of Advanced Sciences, KLE Technological University, Vidyanagar, Hubballi 580 031, Karnataka, India; shanbhagyogesh04@gmail.com (Y.M.S.); shweta.malode@kletech.ac.in (S.J.M.); sdlakshmi97@gmail.com (S.D.); 2 Department of Chemistry, K.L.E. Institute of Technology, Hubballi 580 027, Karnataka, India; maheshshanbhag057@gmail.com; 3 Idaho National Laboratory, Idaho Falls, ID 83415, USA

**Keywords:** graphitic carbon nitride, Pramipexole, voltammetric approach, heterogenous rate constant, diffusion coefficient

## Abstract

Pramipexole (PMXL) belongs to the benzothiazole class of aromatic compounds and is used in treating Parkinson’s disease; however, overdosage leads to some abnormal effects that could trigger severe side effects. Therefore, it demands a sensitive analytical tool for trace level detection. In this work, we successfully developed an electrochemical sensor for the trace level detection of PMXL, using the voltammetric method. For the analysis, graphitic carbon nitride (gCN) was opted and synthesized by using a high-temperature thermal condensation method. The synthesized nanoparticles were employed for surface characterization, using transmission electron microscopy (TEM), X-ray diffraction (XRD), and atomic force microscopy (AFM) techniques. The electrochemical characterization of the material was evaluated by using the electrochemical impedance spectroscopy (EIS) technique to evaluate the solution–electrode interface property. The cyclic voltammetry (CV) behavior of PMXL displayed an anodic peak in the forward scan, indicating that PMXL underwent electrooxidation, and an enhanced detection peak with lower detection potential was achieved for gCN-modified carbon paste electrode (gCN·CPE). The influence of different parameters on the electrochemical behavior was analyzed, revealing the diffusion governing the electrode process with an equal number of hydronium ions and electron involvement. For the fabricated gCN·CPE, good linearity range was noticed from 0.05 to 500 µM, and a lower detection limit (L_D_) of 0.012 µM was achieved for the selected concentration range (0.5 to 30 µM). Selectivity of the electrode in PMXL detection was investigated by conducting an interference study, while the tablet sample analysis demonstrates the sensitive and real-time application of the electrode. The good recovery values for the analysis illustrate the efficiency of the electrode for PMXL analysis.

## 1. Introduction

**Figure 1:**
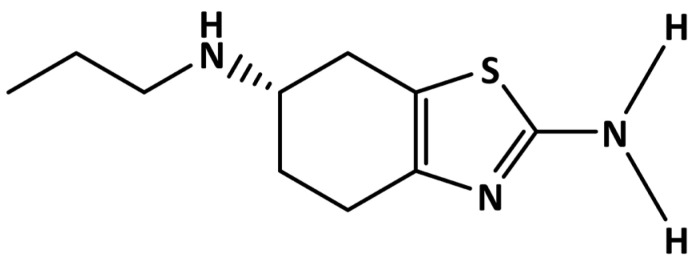
Molecular structure of PMXL.

Pramipexole (PMXL) belongs to the benzothiazole class of aromatic compounds, in which two hydrogens are substituted with an amino group at the 2-pro-S-position and propylamine group substituted at 6-pro-S-position of 4,5,6,7-tetrahydro-1,3-benzothiazole (Figure 1).

Dopamine is secreted by nerve cells, and acts as a neurotransmitter by transmitting signals from one nerve cell to another [1]. Dopamine also plays an important role in maintaining hormonal balance, cardiovascular system, renal system, and metabolism. An abnormal level of dopamine is attributed to Parkinson’s disease (PD) and Schizophrenia disease [2]. The abnormal function of the neurons that secret the dopamine causes a lower level of dopamine, resulting in PD and tremors, rigidity, and bradykinesia [3]. PXML is also used to rat restless leg syndrome.

PMXL has some of the same effects as dopamine and acts as an anti-Parkinson drug, an antidyskinesia, and a radical scavenger. PXML is used for the treatment of PD. It is a non-ergot dopamine agonist at the D2 subfamily of dopamine receptors, with higher selectivity for D3 than for D2 and D4 dopamine receptors [4,5,6], PXML binds to these receptors and mimics the effects of dopamine, which is an efficacious treatment for PD symptoms. PMXL was first proved by the FDA in 1997 for human usage. PXML has an absolute oral bioavailability greater than 90%, indicating good absorption, and its elimination half-life is 8 h [4,7,8]. Serious side effects of PXML are a hallucination, extreme drowsiness, tremors, twitching or uncontrolled muscle movements, vision problems, and uncontrolled posture changes such as involuntary bending forward of the neck. Hence, to monitor the trace level of PXML, new methods must be developed to control the unwanted side effects. 

Electrochemical analytical techniques have recently been extensively explored in fields such as food, pharmaceuticals, healthcare, agriculture, ecology, and textiles [9,10,11,12,13,14,15,16,17]. There are several advantages of electrochemical analysis, such as high sensitivity, selectivity, ease of use, less cost compared to other techniques, and fast analysis [18,19,20]. However, using bare electrodes has a number of disadvantages, such as electrode fouling and poor electron transfer. Modifier intercalation is significantly important in resolving the problems associated with using bare electrodes. Nanomaterials, nanocrystals, and dyes are usually used as modifiers that reduce the excess potential required for the propagation of electrons, increasing the selectivity and sensitivity of an electrode [21,22,23,24]. In recent years carbon paste electrodes have been widely explored in the electrochemical analysis due to their simple fabrication method, can be instantaneously used, and is suitable in a wide range of potential with low background current [21].

Carbon nitride belongs to the class of polymeric materials, consisting mainly of carbon and nitrogen [25,26]. Graphitic carbon nitride (gCN) has a long history, as most carbon materials do. It is considered one of the oldest synthetic compounds. In 1834 Berzelius prepared material named melon and later reported by Liebig [27]. gCN is wonderful material and has gained significant importance in the research field due to the tunable band gap between 1.8 and 2.7 eV with several properties, including low cost, high thermal durability, 2D layered molecular structure with biocompatibility, and stability [28]. Due to its versatile surface properties such as graphite, it is an excellent choice for electrochemical analysis [29]. Due to its tunable bandwidth, it exhibits high electric conductivity, which, in turn, enhances the electron transport in the carbon elements. Moreover, gCN has been utilized in several fields for studies such as photolytic applications [30,31], degradation of organic contaminants [32], biomedical applications [33], hydrogen generation [34], and environmental decontamination [35].

Therefore, the work aims to develop an electrochemical sensor for PMXL detection and determination by using gCN nanoparticles. Furthermore, the carbon-based electrode was modified with the synthesized gCN to develop a new electrochemical sensor. To achieve the best response, electrolyte optimization is carried along with optimization of immersion time. In conclusion, we have proposed a simple, rapid, and cost-effective electrode with sensitivity and selectivity to PMXL. The electrochemical behavior was evaluated by using a voltammetric approach and finally, the efficiency of the electrode was tested with a real sample, and recovery values were recorded.

## 2. Materials and Methods

### 2.1. Used Chemicals and Reagents

Pramipexole dihydrochloride (PXML, assay ≥ 98%) was purchased from Sigma-Aldrich. PXML working standard solutions were prepared in double-distilled water. Series of phosphate buffers (PB) were prepared for electrochemical analysis. PB was prepared with an ionic power (I) of 2.0 × 10^−1^ M, ranging from pH 3.0 to 8.0. Orthophosphoric acid (H_3_PO_4_) (purity ≥ 98%), disodium phosphate (Na_2_HPO_4_) (purity ≥ 97%), trisodium phosphate (Na_3_PO_4_) (purity ≥ 98%), and monopotassium phosphate (KH_2_PO_4_) (purity ≥ 99.5%) are used in phosphate buffer formulation. Carbon paste was prepared from graphite powder (purity ≥ 99.5%) and paraffin oil. Every chemical used in this study was of analytical grade (purity ≥ 97.5%), and for electrochemical investigation, double-distilled water was used.

### 2.2. Synthesis of gCN

**Figure 2:**
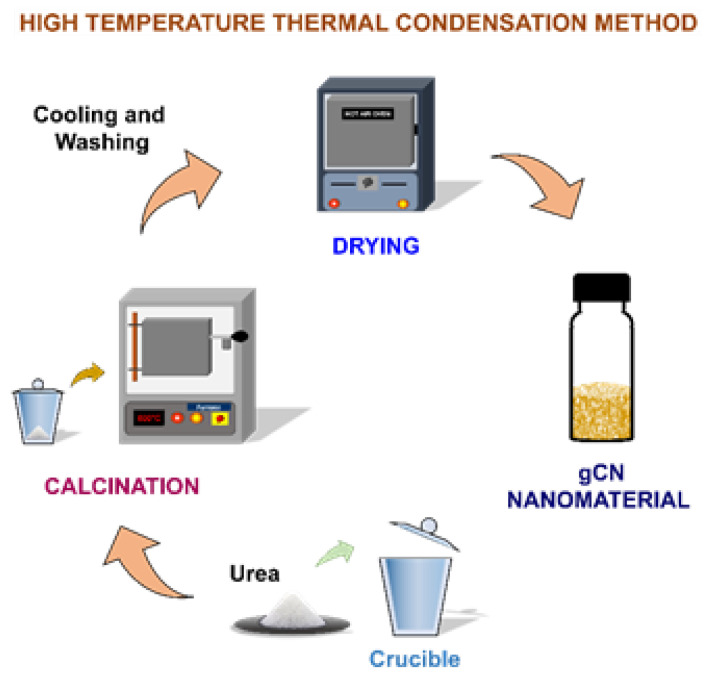
Schematic representation of gCN synthesis.

Nanostructured gCN was synthesized by using the high-temperature thermal condensation method. Then 9.0 g of urea was heated to 600 °C for 4 h; the resulting material was cooled at ambient temperature, washed with double distilled water, and dried. The schematic representation is given in Figure 2.

### 2.3. Equipment and Instruments Used

D630 model, potentiostat (CHI company, Bee Cave, TX, USA) with 3 electrode systems employed in the electrochemical analysis. The working electrode (WE) was gCN revised CPE with the bare electrode as CPE. In the study, a saturated silver–silver chloride electrode was used as a reference electrode (RE). RE was used to measure the changes in potential during electrochemical analysis. A platinum electrode was used as a counter electrode (CE) to complete the circuit. The pH meter (pH-1100 model) from Horiba, Japan, was used to check the pH of the PB. The materials were characterized by using an atomic force microscope (AFM) (Nanosurf AG-easy Scan AFM, Liestal, Switzerland) and X-ray diffraction (powder X-ray diffractometer, Model: SmartLab SE).

### 2.4. Construction of Working Electrode

First, 7.0 g of graphite powder and 3.0 mL of paraffin oil (mineral oil) were mixed in mortar to make a carbon paste. The polytetrafluoroethylene (PTFE) tube with copper wire at one end was filled with homogenized carbon paste. For modified working electrode preparation, 0.05 g of gCN was added to a mortar containing 7.0 g of graphite powder and 3.0 mL of paraffin oil and blended until it became a homogenized mixture. After preparing a smooth homogenized mixture, it was packed in the PTFE tube and used as a gCN·CPE working electrode. To get an even surface and to remove the excessively adhered particles, the electrode was smoothened and washed with double-distilled water. To activate the electrode, an electrolyte of pH 3.02 (PB) was used and was scanned in the fixed potential of 0.2 to 1.4 V, at a scan rate of 0.1 Vs^−1^.

### 2.5. Method of Analysis

A 5.0 mM solution of PXML working standard solution was prepared by dissolving a suitable amount in double-distilled water. The activated electrode was then placed into an experimental cell containing 9.0 mL of PB and 1.0 mL of 5.0 mM PXML solution. CV was recorded by using the defined method at 0.1 Vs^−1^ scan rate. The electrochemical behavior of PXML and the influence of different parameters were studied by CV and SWV. All the electrochemical analyses were performed at 25 (±2) °C.

### 2.6. Formulation of Drug Sample

The PXML tablets were procured from a local pharmacy; 6–10 tablets of PXML were taken in a mortar and crushed until fine tablet powder was obtained, which was used for the preparation of clinical samples. The required quantity of powdered tablets (equivalent to weight of pure analyte) was taken in a 10.0 mL volumetric flask to prepare a 5.0 mM solution of PXML, and then it was diluted with double-distilled water and used as tablet stock solution. The PXML tablet sample solution was sonicated for 10 min to achieve the complete dissolution. The analysis was carried out by adding prepared sample solution into the experimental cell by the conventional addition method.

### 2.7. Interference Study

It is vital to study the interference of the excipients and metal ions in the electrochemical investigation and detection of PXML to check the selectivity of the sensor. To establish the sensor’s selectivity, a number of excipients and metal ion solutions were prepared by dissolving a suitable quantity of excipients and metal ions in double-distilled water to obtain a 0.01 M solution. The requisite aliquots of each excipient or metal ion sample were added to the experimental cell, along with the analyte solution, and an investigation was conducted. 

## 3. Results and Discussions

### 3.1. Characterization of the Electrode Material

**Figure 3:**
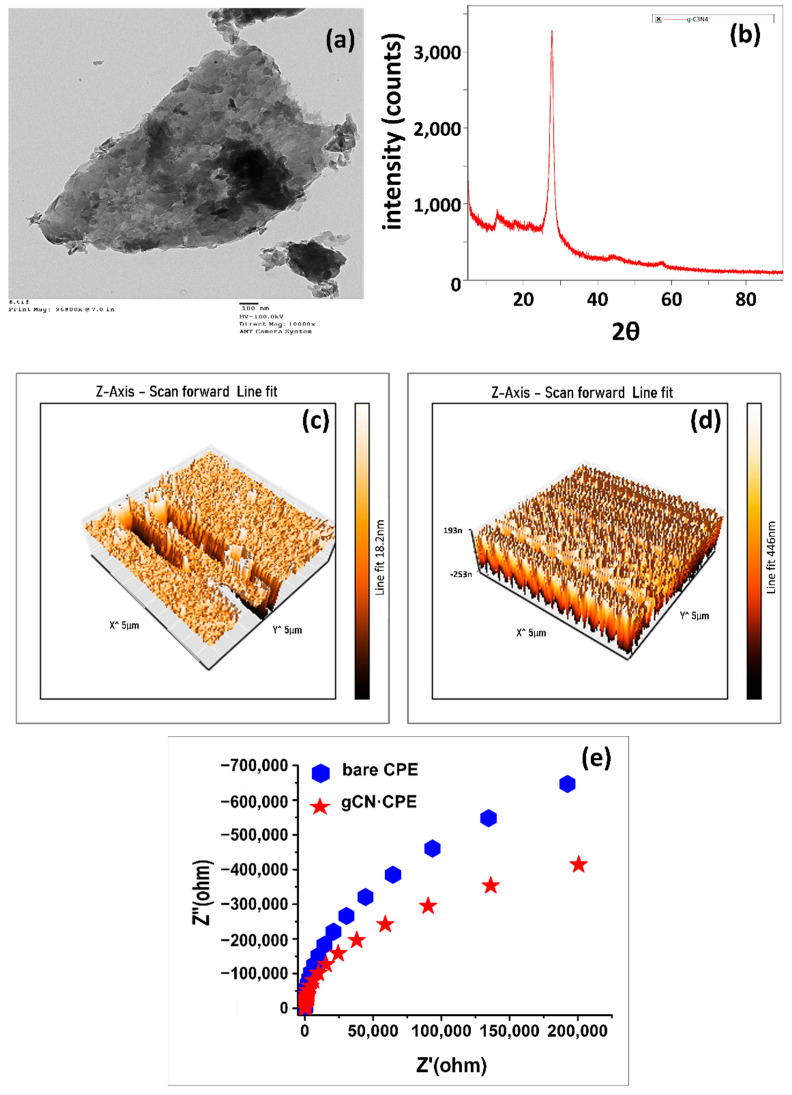
Characterization of electrode material-gCN: (**a**) TEM image, (**b**) XRD image, AFM images of (**c**) CPE, and (**d**) gCN·CPE and EIS study of developed electrodes. (**e**) Nyquist plot.

The TEM analysis of gCN suggests the nanosheet structure of the material, which would be beneficial for the sensing mechanism. The layered and flaky arrangement of the gCN could be the cause for the higher surface area. The TEM image of the gCN nanosheet is depicted in Figure 3a. The XRD study of gCN displayed six diffraction peaks at 2θ values of 12.9°, 21.9°, 27.7°, 29.3°, 43.9°, and 57.2°, respectively (Figure 3b). However, two intense diffraction peaks at 12.9° and 27.7° suggest the interlayer packing and inter-planar stacking of gCN, respectively. The crystallite size of gCN was determined by using the Scherrer equation (Equation (1)), and the average crystallite size of the gCN was calculated to be 11.14 nm.

D = κλ/β·COS(θ)(1)

The surface roughness of the material also influences the sensing interaction; thus, the surface topography of the electrode materials was examined by using the AFM approach. The AFM images of bare CPE and gCN·CPE were shown in Figure 3c,d. The AFM images show the increased surface area of gCN·CPE over bare CPE. The topography shows the uniform conical and planar morphology for gCN·CPE; meanwhile, a non-uniform conical structure was observed for CPE. A uniformly placed conical shape can be attributed to the uniformly deposited and distributed gCN nanosheets [36,37]. This suggests the incidence of more active centers for gCN·CPE than in bare CPE; hence, the surface roughness for both matrices was evaluated and found to be 25.2 pm^2^ for CPE and 25.9 pm^2^ for gCN·CPE, respectively.

To evaluate the ability of the electrode material in electron transfer activity, an EIS investigation was carried out for both electrodes in 5.0 mM PMXL solution, using PBS with a pH of 3.02 as an electrolyte. The EIS study was performed at an amplitude of 0.01 V, within the frequency range of 1.0 to 10^5^ Hz at the electro-oxidation potential of PMXL. The Nyquist plot for electrodes was represented in Figure 2e, and the charge transfer resistance (R_ct_) values were found to be 2366.1 and 451.1 kΩ for CPE and gCN·CPE, respectively. The obtained R_ct_ values suggest the lower conductivity of bare CPE with poor and sluggish electron transfer, whereas gCN·CPE has higher electron transfer efficiency and conductivity.

### 3.2. Voltammetric Response

**Figure 4:**
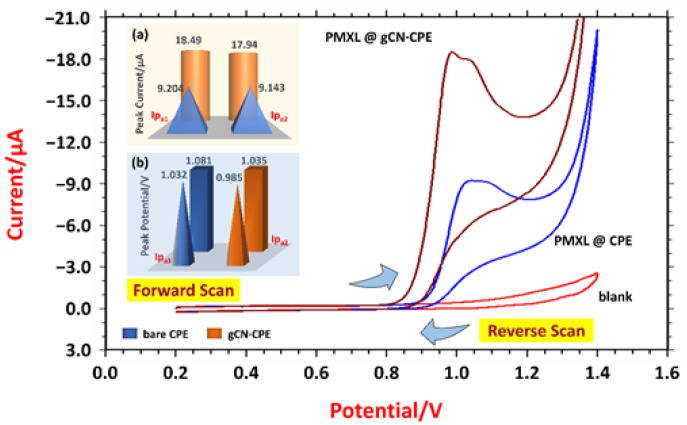
Voltammetric response of 0.5 mM PMXL in PBS of pH 3.02. Comparative bar chart of (**a**) anodic oxidative currents and (**b**) detection potentials.

Cyclic voltammetry (CV) is an effective and widely used electrochemical technique for studying the reduction and oxidation processes of molecular species. Thus, to evaluate the PMXL behavior over the electrode material, the CV technique was employed. The recorded voltammogram (Figure 4) shows the voltammetry response at the electrodes in the absence and presence of PMXL. In the absence of PMXL, no peaks were detected in the forward or the backward scan, and in the presence of PMXL (0.5 mM), both electrodes displayed anodic peaks in the forward scan. No reductive peaks were observed for PMXL in the reverse scan, thus confirming the irreversible electrode process. 

PMXL shows two oxidation peaks for both electrodes; at bare CPE, the peaks were marginally merged; and at gCN·CPE, the two peaks are distinguishable. For CPE, the first oxidation peak was recorded at a peak potential (Ep_a1_) of 1.032 V, with peak current (Ip_a1_) of 9.204 µA, and the second peak was detected at an Ep_a2_ of 1.081 V, with an Ip_a2_ of 9.143 µA. Moreover, due to the catalytic and large surface area of gCN, the modified electrode displayed well-resolved oxidation peaks at a lower detecting potential. For gCN·CPE, the first sharp peak was observed with an Ip_a1_ of 18.49 µA at 0.985 V, and the second peak of 17.94 µA (Ip_a2_) was recorded at 1.035 V. The voltammograms suggest and confirm the higher efficiency of transfer of electron at gCN·CPE, as they display a higher detection current; this could be due to modifier assets. Moreover, the EIS response supports the CV response. Furthermore, out of two oxidative anodic peaks, the first peak was considered for the investigation, as it was sharp and well resolved at the gCN·CPE.

### 3.3. Optimization Study

In an electrochemical analysis of an analyte, the medium of the supporting electrolyte and vicinity concentration of the analyte at the electrode has more influence on the electrochemical behavior of the analyte. Thus, optimizing these parameters is essential to achieve the best response relative to establishing the prioritized criteria.

The detection current is directly proportional to vicinity concentration, which is influenced by the concentration gradient that varies with time. However, in this investigation, the effect of the concentration gradient over current response was recorded at different intervals of time, ranging from 0 to 120 s, using CV. As the accumulation of analyte near the electrode varies with the period of time, it is termed “accumulation time” or “immersion time” (t_imm_). The recorded peak responses at different t_imm_ values are represented in Supplementary Materials Figure S1. It can be noticed that the maximum level of vicinity concentration was achieved at 40 s, as the highest peak current was recorded at this particular time interval. The decay in the peak current after 40 s suggests that the longer accumulation time could induce an equilibrium between the diffusion and adsorption layer of PMXL concentration at the interface. A nearly constant peak current was observed with an increasing time interval (from 100 s), indicating the saturation level of PMXL in the electroactive surface covering the gCN·CPE. Thus, a t_imm_ of 40 s has opted for further investigation.

**Figure 5:**
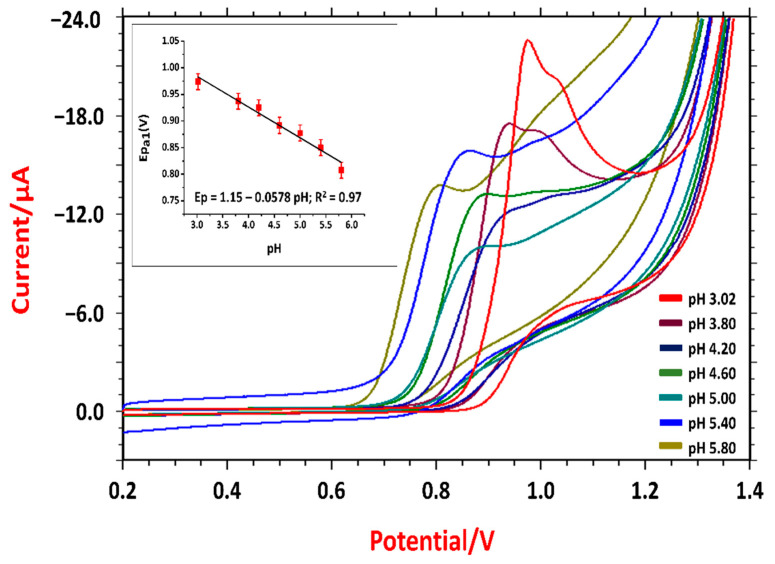
Voltammetric response of 0.5 mM PMXL at various pH values, at t_imm_ of 40 s; dependency of peak potential on pH of PBS.

During the electrode process, there could be possible transfers of protons that can affect the pH, as well as the rate of electro-oxidation reaction steps. The change in magnitude corresponds to a deviation in the acid and base dissociation constants. Thus, the influence of the pH on the oxidative peaks and detection potential was carried out in the acidic range of pH from pH 3.02 to 5.80, using the CV approach. The obtained voltammograms (Figure 5) suggest the dependency of the pH over the voltammetry behavior of PMXL as the detection potential shifted negatively with the increased pH. At a lower acidic medium, two peaks were displayed for PMXL, whereas, with the increased pH, the peaks were merged and exhibited one oxidative peak, indicating the importance of the pH of electrolyte in the electrochemical reaction.

In the electrochemical sensing of PMXL, a pH of 3.02 was perceived to be prominent for the mechanism, as it unveiled the highest detection current; thus, the same has opted for the further electrochemical investigation. 

The negative deviation in the detection potential with increased pH indicates the involvement of the hydronium ion. Thus, the impact of pH on the detection potential was evaluated by plotting pH vs. Ep_a_, as represented in the Figure 5a. The linear relationship was noticed with the regression equation as E_p_ = 1.15–0.0578 pH; R^2^ = 0.97. The experimental slope value is closer to the theoretical Nernst value (0.059 V/pH), thus confirming the participation of two numbers of electrons in the hydronium-ion-coupled reaction mechanism [38]. Furthermore, the number of hydronium ions involved in the electrode process was determined by using the Nernst equation (Equation (2)). The number of hydronium ions that participated in the process was calculated to be 1.95 ≈ 2.

E_p_ = E° + (0.0591/n) log [(ox)^a^/(R)^b^] − (0.0591·m/n) pH (2)

### 3.4. Probable Electrochemical Sensing Mechanism of PMXL at gCN·CPE

Electrode sensing mechanism: From the study on the influence of the sampling rate, it was found that the surface-mediated mechanism of the irreversible mechanism was governed by diffusion. The concentration gradient induces the migration of PMXL from the bulk solution to the electrode surface and undergoes the electrochemical reaction. 

The band gap of the modifier plays a fundamental role in electrical conductivity. The electrical conductivity increases with the decreasing band gap [11]. When a voltage is applied from the external source, it induces the change in the Fermi level of the electrode, resulting in the transfer of electrons. The energy levels of the electrode and the analyte determine the electron transfer between them. Therefore, the electrochemical reaction can be induced by changing the energy level at the electrode [14].

**Scheme 1:**
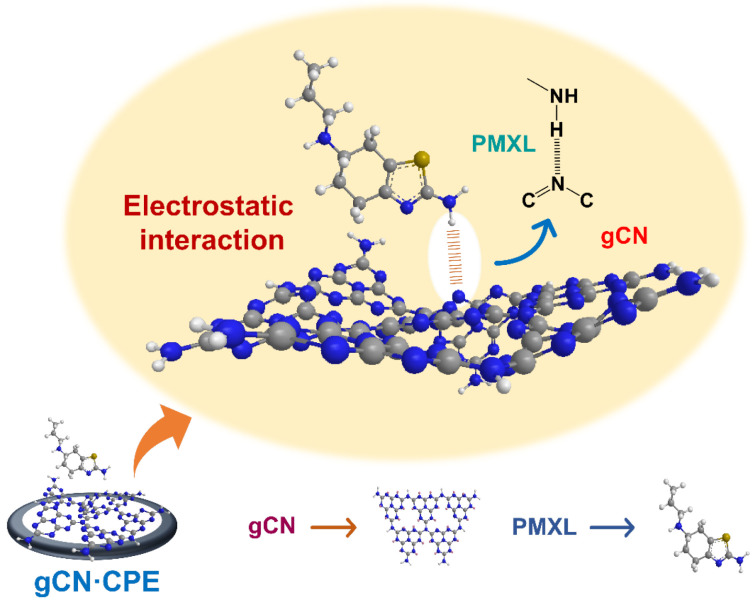
Probable electrochemical sensing mechanism of PMXL at gCN·CPE.

In gCN, carbon and nitrogen atoms are arranged in a honeycomb structure, resulting in a large surface area. The hexagonal rings of gCN are connected with sp^2^ hybridization. In addition, PMXL physisorption was mainly driven by the dispersion interaction; however, electrostatic interaction between the hydrogen of the amine group (PMXL) and the nitrogen of gCN leads to the adsorption of PMXL onto gCN (Scheme 1). This intermolecular bond is sufficient to keep the PMXL in the interface, thus further aiding the electrode process.

### 3.5. Feasible Reaction Mechanism of PMXL at gCN·CPE

**Scheme 2:**
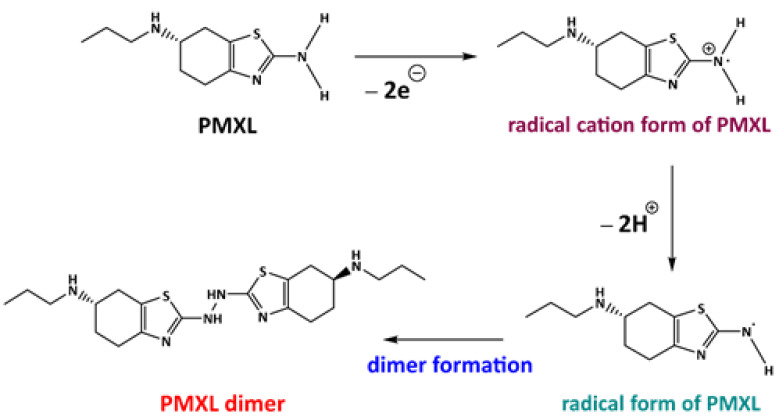
Feasible electrochemical reaction of PMXL at gCN·CPE.

The voltammetric behavior of PMXL at the gCN·CPE displays anodic peaks in the forward scan, and no peak was observed in the backward scan, thus confirming the irreversible system for the electrode process. PMXL is with a functional group that has a higher tendency toward electron donation (electron-donating groups), such as amine, an alkyl group that could undergo electrooxidation under the influence of applied potential. In the PMXL molecule, the primary reaction was initiated by electron eradication from the terminal -NH_2_ group [39] that leads to an unstable radical cation intermediate state. To gain stability, the hydronium ion is removed and forms a free radical, which further reacts together and forms the dimer [40], which is more stable than the monomers. This could be the reason for the irreversibility of the electrode process. The impact of electrolytes revealed the involvement of an equal number of electrons and hydronium ions in the process. The proposed reaction mechanism is shown in Scheme 2.

### 3.6. Impact of Scan Rates on PXML

The scan rate is an important electrochemical parameter in the voltammetry study; the change in scan rate triggers the deviation in current and potential. The impact of the scan rate on the PMXL’s behavior was determined by the CV approach by varying the scan rate from 0.01 to 0.27 Vs^−1^, at a t_imm_ of 40 s. The recorded CV was given in Supplementary Materials Figure S2, and it was noticed that the peak current enhanced with the increased scan rate; this could be due to the increased faradaic current, along with the charging current at higher scan rates. The increased faradaic current would be the result of the increased flux concentration at the electrode, suggesting the linear relationship between the scan rate and PMXL concentration. Thus, a scan rate study would help in the evaluation of kinetics and physicochemical parameters of the electrochemical process.

The heterogeneous rate constant (k°) of the electrode process can be determined by investigating the relationship between the detection potential and log (scan rate). Using Laviron’s equation (Equation (3)) [41], the k° and the charge transfer coefficient (α) value for the irreversible process can be calculated, respectively. The linear relationship between detection potential and scan rate was achieved as shown in the Supplementary Materials Figure S2, with a regression of Ep_a_ = 0.021 log(ν) + 1.011; R^2^ = 0.99.

E_pa_ = E° + (RT/(1 − α) nF)·ln((1 − α)nF/RTk°) + (RT/(1 − α)nF)·lnν (3)

where E° stands for standard electrode potential and can be determined by the plot of Ep_a_ versus the scan rate. The α value is calculated to be 0.48 and is closer to 0.5, which is ideal for the electrochemical irreversible process. The k° for the oxidation reaction was found to be 10.02 cm·s^−1^.

From the investigation, it was noticed that the square root of scan rate is directly proportional to the peak current (Supplementary Materials Figure S2B). Moreover, the deviation in the detection potential value increases with the increased scan rate, confirming the irreversibility. Furthermore, the dependency of log(ν) on log (Ip_a_) was examined (Supplementary Materials Figure S2C), and the obtained linear equation was as follows: log(Ip_a_) = 0.492 log(ν) + 1.809; R^2^ = 0.96. The obtained slope value was closer to 0.5, suggesting that the electrode process was governed by the diffusion process [42]. Subsequently, the diffusion coefficient for the electrode process was calculated by using the Randles–Sevcik equation (Equation (4)) [43] and found to be 3.13 × 10^−11^ cm^2^·s^−1^.

I_p_ = (2.99 × 10^5^)·n·(αn_a_)^1/2^·A°·D^1/2^·C*·ν^1/2^ (4)

where n_a_ represents the number of electrons that participated in the rate-determining step; in this case, n_a_ was 1. A° stands for the active surface area of the electrode (it was calculated to be 0.0509 cm^2^ for gCN·CPE). Furthermore, the surface coverage of PMXL over electrodes was computed by using (Equation (5)) [44], and the value was determined to be 7.2 × 10^−8^ mol·cm^−2^.

I_p_ = n^2^ F^2^ A° Γ ν/4RT (5)

where Γ stands for the surface coverage of PMXL on the electrode, A° is the active surface area of the electrode, and the remaining notations have their standard meanings.

### 3.7. Quantification of PMXL Using gCN·CPE

**Figure 6:**
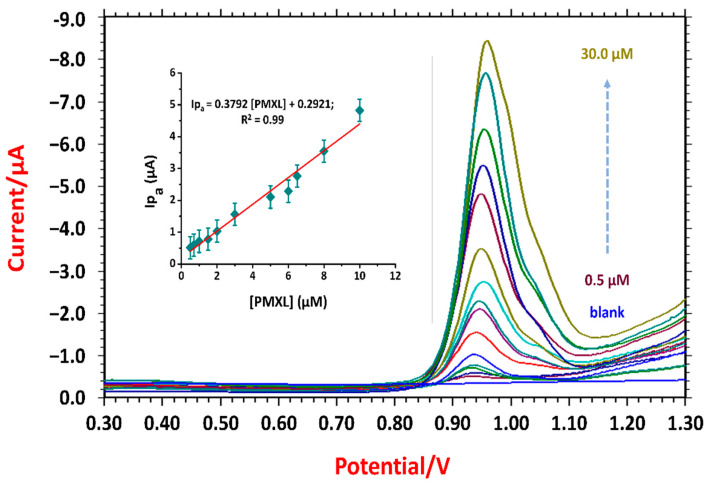
SWV responses of PMXL at different concentrations, at a pH of 3.02, at t_imm_ of 40 s; calibration curve in a tested concentration range.

The fabricated gCN·CPE was tested for sensitivity by varying the concentration from lower (10^−7^ M) to higher concentration (10^−5^ M), using the SWV technique, at a pH of 3.02 and at a t_imm_ of 40 s. The recorded voltammograms are shown in Figure 6. The magnitude of the oxidative peak current was directly proportional to the concentration; therefore, a calibration curve was plotted (Figure 6a). The PMXL concentration displayed linear regions for lower concentrations, and gCN·CPE was employed for the determination of limit of detection (L_D_) and limit of quantification (L_Q_). The detection limit (L_D_) and quantification limit (L_Q_) were calculated by using the equations 3 × si/m and 10 × si/m. The linear regression equation for the lower concentration range was obtained as Ip_a_ = 0.3649 [PMXL] + 0.328; R^2^ = 0.99. The values of L_D_ and L_Q_ were found to be 0.012 µM and 0.039 µM, respectively. The characteristics of the calibration curve are given in Supplementary Materials Table S1. These values were contrasted with earlier reported analytical methods and electrodes [40,45,46,47,48,49,50] (Table 1), and it was noticed that the developed electrode was sensitive, convenient, and efficient in PMXL sensing and detection.

### 3.8. Diagnostic Analysis

To check the efficiency and real-time application of the developed electrode, a tablet analysis was carried out. The stock solution was prepared by dissolving a finely powdered table sample (weight equivalent to standard solution). The known volume of the sample aliquots was transferred to the voltammetric cell and tested for the recovery value. The obtained results are given in Table 2, and the recovery values were achieved in the range of 95.58 to 96.24%, with a % RSD of 0.90.

### 3.9. Interference Study 

During the tablet formulation, some additives were added to enhance the stability, dissolution rate, disintegration, and bioavailability, and these additives are generally known as excipients. There would be a possibility of influence of these excipients on the electrochemical behavior of PMXL; thus, some excipients were tested for interference. Cellulose, gum acacia, mannitol, starch, and titanium dioxide were analyzed in the present investigation, and interference responses were recorded in the predetermined potential window. Furthermore, the change in the % signal was calculated and plotted against the excipients (Supplementary Materials Figure S3), showing the uninterrupted PMXL responses in relation to their detection potential, which qualitatively indicates the selectivity of the electrode. 

Analogously, the effect of different metal ions was investigated; since trace levels of metal ions are present in humans as micronutrients, that may lead to the formation of the complex. Thus, some of the communal metal salts were spiked and tested for interference. The obtained voltammograms show that (Supplementary Materials Figure S4) the deflection in the peak potential was within the ±5%, indicating that the metal ion did not interfere in the analysis to a greater extent, supports the selectivity of the electrode. The details of the interference study are given in Table 3.

### 3.10. Stability of the Electrode

The determination of stability of the electrode material in the sensing process is necessary to evaluate its capacity and capability in electrochemical analysis. The repeatability and reproducibility of the gCN·CPE was tested by observing its response to PMXL within the set time. The electrode matrix was stored in an airtight container for two weeks, and then it was used to fabricate the electrode, and the PMXL (0.05 mM) response was recorded. The obtained voltammograms were compared with the previously obtained response and found to be 92.82–96.85% retention of the original peak and potential. This demonstrates the repeatability of the sensing material.

Similarly, the response for PMXL (0.05 mM) was recorded within a day with a set interval of time. The voltammograms displayed a similar behavior with the same current magnitude at the desired potential with 2.24% of RSD (three replicates). This indicates the high reproducibility of the electrode. The CV behavior for stability investigation is given in (Supplementary Materials Figure S5). The details of the stability are depicted in Table 4.

## 4. Conclusions

A new simple, rapid, cost-effective electrode was developed by using gCN nanoparticles for the direct electrochemical analysis of PMXL. The gCN was synthesized by the high thermal conduction method, and it was implanted in the electrode modification. The synthesized gCN was tested for TEM, XRD, AFM, and EIS to check the surface morphology and crystallinity. The average crystallite size of gCN was calculated to be 11.14 nm. The EIS study revealed the high conductivity and low resistance for the gCN·CPE, as confirmed by CV analysis. The effects of the pH and accumulation time were optimized. The scan-rate study revealed the diffusion-governed electrooxidation process involving two hydronium ions and electrons, respectively. The electrochemical process was prominent in the PBS with a pH of 3.02, and the dependency of detection potential on pH confirms the hydronium ion involvement in the electrode process. The heterogeneous rate constant was determined to be 10.02 cm·s^−1^, with the diffusion coefficient of 3.13 × 10^−11^ cm^2^·s^−1^. The electrochemical behavior was excellent at the gCN·CPE and was evaluated in the concentration range of 0.5 to 30.0 µM, and the L_D_ was calculated to be 0.012 µM with good reproducibility. The tablet analysis demonstrates the efficiency of the developed electrode toward the real-time application, whereas the interference study illustrates the selectivity of the electrode. Finally, the developed sensor offers many advantages of sensitive and selective detection within the dynamic concentration range with higher stability and reproducibility.

## Figures and Tables

**Table 1:** Comparative statement with pre-reported methods.

Comparative Study				
Methods/Sensors Utilized	Linearity Range (µM)	L_D_ (µM)	Sensitivity (µA·µM^−1^·cm^−2^)	Reference
UV	0.04 to 1.4	0.015	-	[45]
Capillary electrophoresis	0.2 to 3.5	0.04	-	[46]
HPLC	0.02 to 1.4	0.01	-	[47]
ZSM-5 nanozeolite and TiO_2_ nanoparticles	0.6 to 105.0	0.38	0.44	[40]
Carbon nanotube–modified glassy carbon electrodes	0.013 to 0.66	0.023	-	[49]
Titanium dioxide nanoparticles–modified carbon paste electrode	0.46 to 100	0.14	0.66	[50]
Graphitic carbon nitride–modified carbon paste electrode	0.05 to 500	0.012	7.44	Present work

**Table 2:** Details of PMXL analysis in pharmaceutical drug samples.

Drug Spiked	Tablet Samples	Spiked (10^−6^ M)	Detected * (10^−6^ M)	% Recovery	RSD	% RSD
PMXL	Aliquot-1	5.0	4.78	95.58	0.0092	0.91
	Aliquot-2	10.0	9.73	97.32	0.0090	0.89
	Aliquot-3	15.0	14.44	96.24	0.0091	0.90

* Average of five determinations.

**Table 3:** Details of interference response of PMXL at gCN·CPE.

Interferent	Detected E_pa_ (V)	Standard E_pa_ (V)	Change in % E_pa_
Excipients			
Cellulose	0.94	0.948	−0.84
Gum acacia	0.944	0.948	−0.42
Mannitol	0.944	0.948	−0.42
Starch	0.936	0.948	−1.26
TiO_2_	0.96	0.948	1.26
Metal Ions			
Ca(NO_3_)_2_	0.936	0.948	−1.26
CuSO_4_	0.94	0.948	−0.84
FeSO_4_	0.956	0.948	0.84
KCl	0.944	0.948	−0.42
MnSO_4_	0.944	0.948	−0.42
NaCl	0.952	0.948	0.42

PMXL = 1.0 × 10^−5^ M, excipient = 1.0 × 10^−4^ M, and metal ions = 1.0 × 10^−4^ M.

**Table 4:** Details of stability of the electrode.

Reproducibility					
CV Responses	Detected Response	Original Response	Response Retention %	RSD	% RSD
At 1st day	22.57	22.57	100.00	0.031	3.14
At 4th day	22.57	21.86	96.85	0.032	3.24
At 8th day	22.57	21.29	94.33	0.033	3.33
At 12th day	22.57	20.95	92.82	0.033	3.38
% Retention: 92.82–96.85; Average % retention: 96.01; % RSD: 3.14					
Repeatability					
At 0 h	22.57	22.57	100.00	0.021	2.18
After 12 h	22.57	21.76	96.41	0.022	2.26
After 24 h	22.57	21.68	96.06	0.022	2.27
% Retention: 96.06–100.0; Average % retention: 97.5; % RSD: 2.24					

## Data Availability

Not applicable.

## Supplementary Materials

The following supporting information can be downloaded at: https://www.mdpi.com/article/10.3390/bios12080552/s1. Figure S1: Effect of immersion time; Figure S2: (A) Voltammetric behavior of 0.5 mM PMXL at different scan rates in pH 3.02 (inset: (a) influence of scan rate of peak potential); (B) dependency of peak current on square root of scan rate; (C) realtionship be-tween log(v) and log(Ip); Figure S3: SWV responses of PMXL in presence of different excipients in pH 3.02 at timm of 40 s; (a) Bar diagram for change in % signal; Figure S4: SWV of metal ion interference study inset: (a) graphical representation for % signal change; Figure S5: Electrode stability investigation. CV response of (A) Repeatability; (B) Reproducibility; Table S1: Specifications of calibration curve of PMXL at gCN·CPE.
