# Supplementary material for: Direct and Sensitive Electrochemical Evaluation of Pramipexole Using Graphitic Carbon Nitride (gCN) Sensor
Source: Biosensors (Basel). 2022 Jul 22;12(8):552. doi: 10.3390/bios12080552 (PMC9394362; doi:10.3390/bios12080552)
Supplement: Supplementary file 1 [file biosensors-12-00552-s001.zip › biosensors-1818319-supplementary-done.pdf]

biosensors

Direct and sensitive electrochemical evaluation of pramipexole using graphitic carbon nitride (gCN) sensor

Yogesh M. Shanbhag[1], Mahesh M. Shanbhag[2], Shweta J. Malode[1], S. Dhanalakshmi[1], Kunal Mondal[3]*, Nagaraj P. Shetti[1]*

Supplementary Information

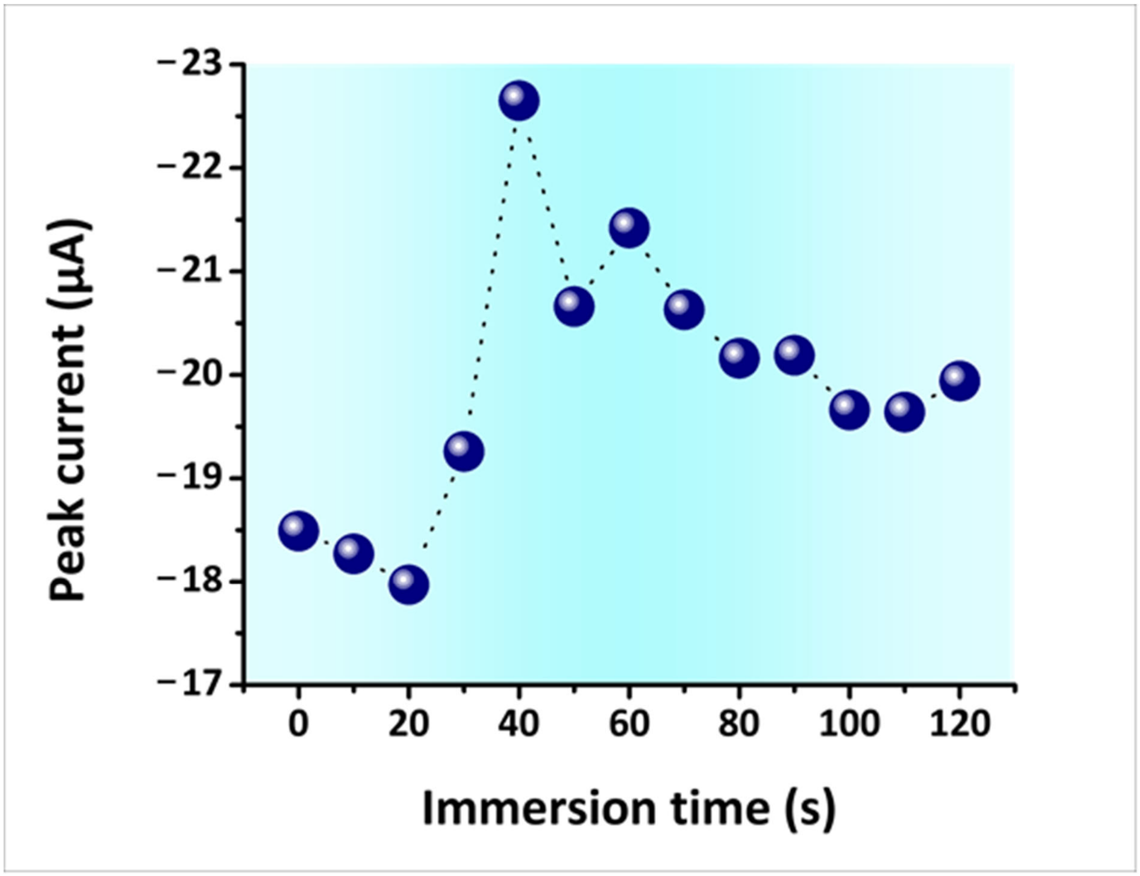

Figure S1. Effect of immersion time.

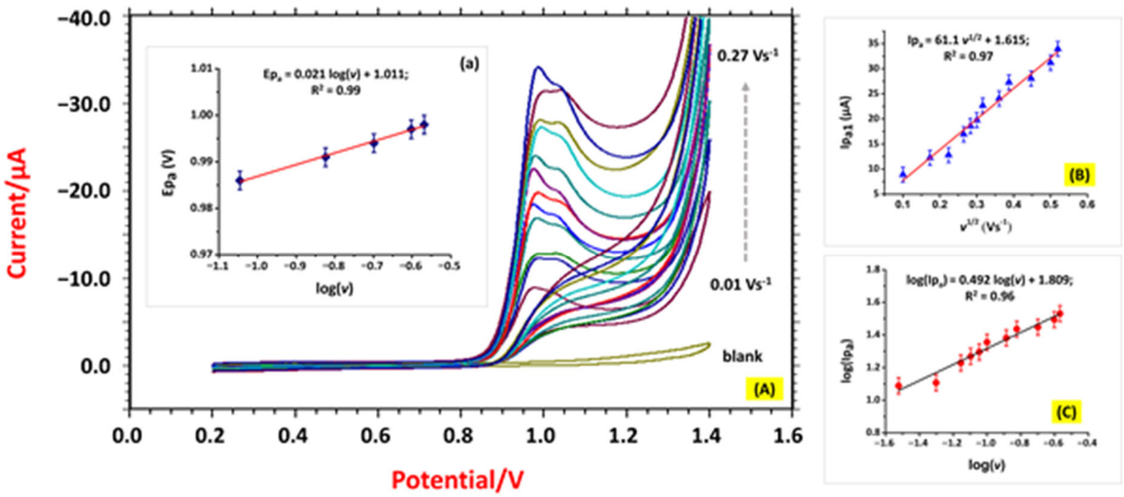

Figure S2. (A) Voltammetric behavior of 0.5 mM PMXL at different scan rates in pH 3.02 (inset: (a) influence of scan rate of peak potential); (B) dependency of peak current on square root of scan rate; (C) realtionship between log(v) and log(Ip).

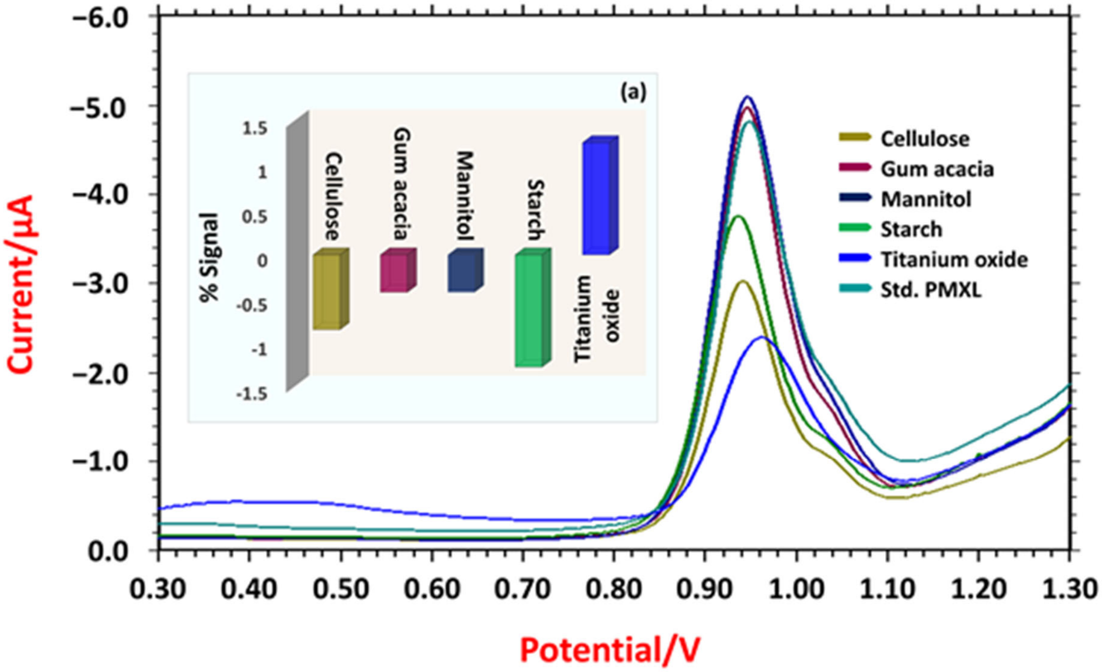

Figure S3. SWV responses of PMXL in presence of different excipients in pH 3.02 at t_{imm} of 40 s; (a) Bar diagram for change in % signal.

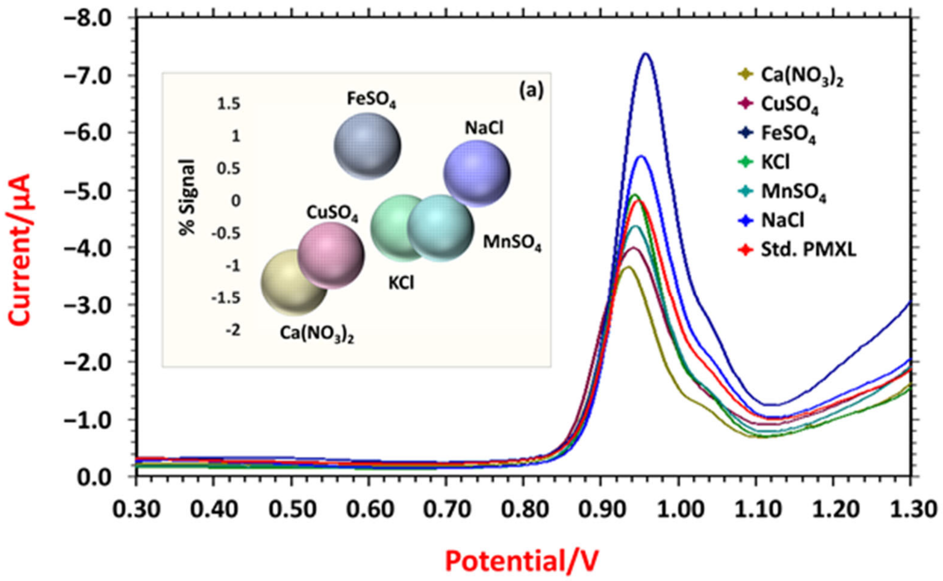

Figure S4. SWV of metal ion interference study inset: (a) graphical representation for % signal change.

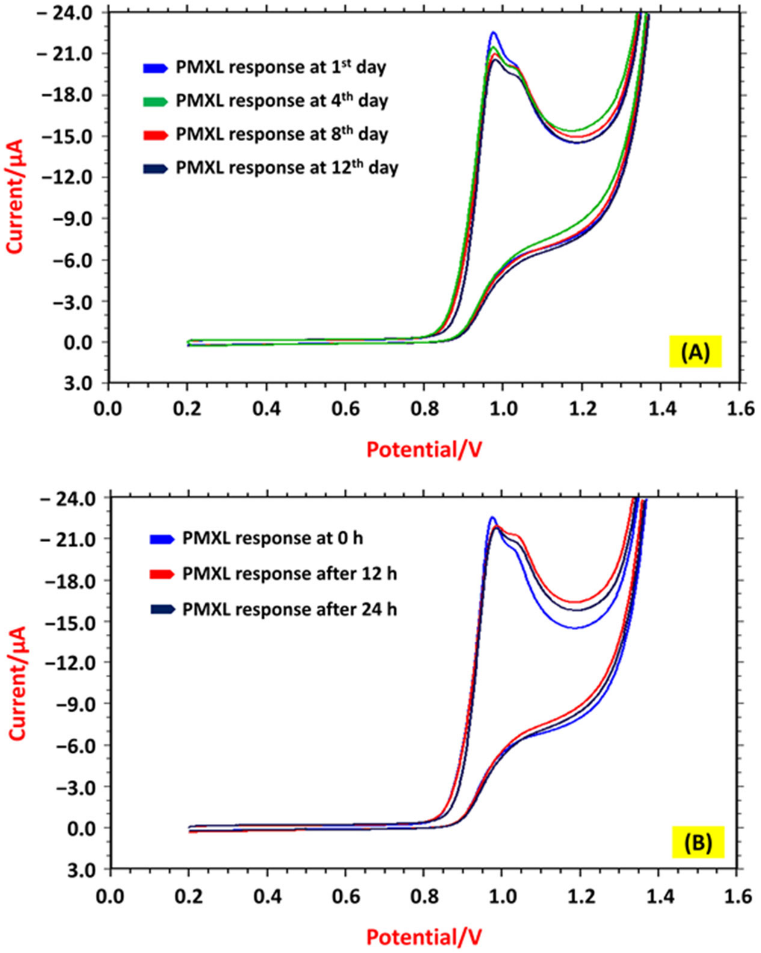

Figure S5. Electrode stability investigation. CV response of (A) Repeatability; (B) Reproducibility.

Table S1. Specifications of calibration curve of PMXL at gCN·CPE.

Linearity (μM)	0.05 – 500.0
Calibration curve slope (μA M^{-1})	0.379
Intercept of calibration curve (μA)	0.292
SD of intercept	0.00517
Average slope value	0. 3798
Regression coefficient (R^2)	0.99
RSD of the slope (in %)	0.385
RSD of the intercept (in %)	0.520
Total test point considered	11
L_D (μM)	0.012
L_Q (μM)	0.039
Sensitivity (μA·μM^{-1}·cm^{-2})	7.44
% RSD for repeatability	1.661
% RSD for reproducibility	0.790
